# Plasma Membrane Profiling Reveals Upregulation of ABCA1 by Infected Macrophages Leading to Restriction of Mycobacterial Growth

**DOI:** 10.3389/fmicb.2016.01086

**Published:** 2016-07-12

**Authors:** Jing Long, Robindra Basu Roy, Yanjia J. Zhang, Robin Antrobus, Yuxian Du, Duncan L. Smith, Michael P. Weekes, Babak Javid

**Affiliations:** ^1^Collaboration Innovation Centre for the Diagnosis and Treatment of Infectious Diseases, School of Medicine, Tsinghua UniversityBeijing, China; ^2^Harvard TH Chan School of Public Health, BostonMA, USA; ^3^Cambridge Institute for Medical Research, University of CambridgeCambridge, UK; ^4^Cancer Research UK Manchester Institute, University of ManchesterManchester, UK

**Keywords:** plasma membrane profiling, *Mycobacterium*, ABCA1, cholesterol, SILAC

## Abstract

The plasma membrane represents a critical interface between the internal and extracellular environments, and harbors multiple proteins key receptors and transporters that play important roles in restriction of intracellular infection. We applied plasma membrane profiling, a technique that combines quantitative mass spectrometry with selective cell surface aminooxy-biotinylation, to Bacille Calmette–Guérin (BCG)-infected THP-1 macrophages. We quantified 559 PM proteins in BCG-infected THP-1 cells. One significantly upregulated cell-surface protein was the cholesterol transporter ABCA1. We showed that ABCA1 was upregulated on the macrophage cell-surface following infection with pathogenic mycobacteria and knockdown of ABCA1 resulted in increased mycobacterial survival within macrophages, suggesting that it may be a novel mycobacterial host-restriction factor.

## Introduction

*Mycobacterium tuberculosis* (Mtb) is a human intracellular pathogen that causes tuberculosis (TB) – a major global disease with 9.0 million new cases and 1.5 million deaths a year. Human beings have been the only natural niche for *M. tuberculosis* for tens of thousands of years (Comas et al., 2013), and therefore Mtb is highly adapted to its host environment. A better understanding of how Mtb engages in its intracellular lifestyle, and of specific host responses to infection will be vital to inform both host-directed therapies (Stanley et al., 2014; Zumla et al., 2015) and vaccine design.

A variety of proteomic approaches have been applied both to mycobacteria (Jungblut et al., 1999; de Souza et al., 2010; de Souza and Wiker, 2011; Schubert et al., 2015), and cells stimulated by infection (Rao et al., 2009; Wang et al., 2013) or mycobacterial bioactive lipids (Shui et al., 2009). Given the intracellular niche within the macrophage that both the vaccine strain *M. bovis-*BCG (BCG) and Mtb occupy, a key part of understanding how infection interacts with host innate and adaptive immunity is to define how proteins are modulated on the surface of infected cells. This interface between the intracellular and extracellular environment is fundamental to cellular functions such as cell signaling and nutrition, and therefore a rich source for investigating both host defenses and pathogenic mechanisms.

Plasma membrane profiling (PMP) is an unbiased proteomic approach that combines stable isotope labeling by amino acids in cell culture (SILAC)–based differential analysis with selective enrichment of plasma membrane (PM) proteins through selective oxidation and aminooxy-biotinylation of sialylated PM glycoproteins (Weekes et al., 2010, 2012). We have employed PM profiling to identify a cell surface marker of latent human cytomegalovirus infection, enabling novel approaches to therapy and diagnosis (Weekes et al., 2013). We applied this technique for the first time to bacteria-infected human cells to investigate host-pathogen interactions. Through focusing specifically on PM proteins, we present an example of the study of a specific organelle during the course of mycobacterial infection (Rao et al., 2009; Shui et al., 2009; Li et al., 2011; Jamwal et al., 2013; Wang et al., 2013; Saquib et al., 2015).

We quantified 559 PM proteins in BCG-infected macrophages. We used forward- and reverse-labeled SILAC to identify ‘high confidence’ hits, which included proteins involved in the innate and adaptive immune systems, adhesion, and cholesterol metabolism. The cholesterol transporter ABCA1 was significantly upregulated in response to mycobacterial infection and restricts mycobacterial growth – thus upregulation of ABCA1 appears to be a novel host restriction factor for mycobacterial infection.

## Materials and Methods

### Cells and Cell Culture

Human monocytic cells (THP-1) were cultured at 37°C in SILAC RPMI-1640 medium (Thermo Pierce) supplemented with 10% dialyzed fetal calf serum (FCS). Media was supplemented with either light (Arg 0, Lys 0, Fisher Scientific) or heavy (Arg 10, Lys 8, Fisher Scientific) amino acids at 50 mg/L and L-proline at 200 mg/L. The incorporation of heavy label was checked by analysis of a lysate of 3 × 10^6^ heavy labeled cells generated using 4% Sodium dodecyl sulfate (SDS)/Dithiothreitol (DTT)/Tris buffer and Filter Aided Sample Processing (FASP) (Wisniewski et al., 2009). Incorporation was >97% for both arginine and lysine-containing peptides. Bacille-Calmette–Guerin Pasteur *1173P2* strain (BCG) was grown in 7H9 medium (Difco) supplemented with oleic acid, albumin, dextrose, and catalase (OADC), glycerol and 0.025% Tween-20. BCG was grown to early log phase (OD_600_ ∼0.4) prior to infection of macrophages. BCG expressing green fluorescent protein (BCG-GFP) was constructed by transforming BCG with the episomal plasmid pMV261-GFP (Stover et al., 1991) and selecting for kanamycin resistance. All experiments with BCG and *M. abscessus* (local clinical isolate, kind gift from Dr. Hairong Huang, Beijing Tuberculosis and Thoracic Tumor Institute) were carried out in Biosafety level-2 containment facilities according to local guidelines and all experiments with *M. tuberculosis* (strain H37Rv) in a BSL-3 facility according to local guidelines.

### Infection with BCG

THP-1 cells were activated and differentiated into adherent macrophages by overnight incubation with phorbol-12-myristate-13-acetate (PMA) at a concentration of 5 ng/ml prior to infection. Cells were washed and adherent cells infected with BCG. BCG cultures in logarithmic growth phase were centrifuged, resuspended, sonicated, filtered through a 5 μm filter and diluted in serum free SILAC RPMI to achieve a multiplicity of infection of 5:1. Cells were incubated with BCG for 4 h at 37°C and then washed three times with phosphate buffered saline (PBS) to remove extracellular bacteria still in suspension. Adherent control cells underwent the same PMA activation and media changes with PBS washes, but did not undergo infection. For experiment A, light-labeled cells were infected with BCG and heavy-labeled cells were the control. For experiment B, heavy-labeled cells were infected, and light-labeled cells were the control as a ‘label-swap’.

### Preparation of PM Proteins using Aminooxy-Biotin

Plasma membrane profiling was performed as described previously (Weekes et al., 2010, 2012), with minor modifications. Briefly, after 48 h, uninfected and infected adherent cells were scraped, resuspended, and mixed 1:1, using 5.6 × 10^7^ of each cell type. Cells were washed twice with ice-cold PBS. Sialic acid residues were oxidized with sodium meta-periodate (Thermo) then biotinylated with aminooxy-biotin (Biotium). The reaction was quenched, and the biotinylated cells resuspended in 1% Triton X-100 lysis buffer. Biotinylated glycoproteins were enriched with high affinity streptavidin agarose beads (Pierce) and washed extensively. Captured protein was denatured with DTT, alkylated with iodoacetamide (IAA, Sigma) and digested overnight on-bead with trypsin (Promega) in 50 mM ammonium bicarbonate pH at 37°C. For experiment B, ten percent of the resultant digest was desalted and concentrated by StageTip (Rappsilber et al., 2007) for immediate analysis. For experiment A, 90% of the tryptic peptide sample was fractionated by HpRP-HPLC (see below). For both experiments, beads were further washed, and incubated overnight with Peptide-*N*-glycosidase (PNGase) at 37°C in G7 buffer. Glycopeptides were collected, beads were washed once with G7 buffer, and eluates pooled and concentrated on a StageTip (Rappsilber et al., 2007). Peptides were enriched and desalted using StageTips.

### High pH Reverse-Phase High Pressure Liquid Chromatography (HpRP-HPLC) Fractionation and Mass Spectrometric Analysis

High pH Reverse-phase High Pressure Liquid Chromatography (HpRp-HPLC) was performed as described previously (Weekes et al., 2012), using 90% of the tryptic peptide sample from experiment A with a Dionex Ultimate 3000 powered by an ICS-3000 SP pump with an Agilent ZORBAX Extend-C18 column (4.6 mm × 250 mm, 5 μm particle size). Mobile phases (H20, 0.1% NH4OH or MeCN, 0.1% NH4OH) were adjusted to pH 10.5 with the addition of formic acid and peptides were resolved using a linear 40 min 0.1%-40% MeCN gradient at pH 10.5. Eluting peptides were collected in 15 s fractions. Fractions were dried down using an Eppendorf Concentrator and resuspended in 8 μL MS solvent (3% MeCN, 0.1% TFA). Fractions 24 to 136 inclusive were analyzed and in each case 3 μL was injected and subjected to LC-MS/MS using a NanoAcquity uPLC (Waters, MA, USA) coupled to an LTQ OrbiTrapXL (Thermo, FL, UA). Peptides were eluted using a gradient rising from 10 to 25% MeCN by 25 min, 40% MeCN by 28 min and 85% MeCN by 29 min. MS data was acquired between 300 and 1600 m/z at 60,000 FWHM with lockmass enabled (445.120025 m/z). CID spectra were acquired in the LTQ with MSMS switching operating in a top 6 DDA fashion triggered at 500 counts. For both experiments, the glycopeptide samples were eluted using a gradient rising from 3 to 25% MeCN by 90 min, 40% MeCN by 100 min and 85% MeCN by 102 min. Spectra were acquired using the same MS parameters.

### Database Searching and Data Processing

Raw MS files were processed using MaxQuant version 1.3.0.5 (Cox et al., 2009, 2011). Raw files from the glycopeptide samples were searched in parallel with tryptic samples. An experimental design was specified such that all raw files from Experiments A and B were analyzed together, with data output separated within the same spreadsheet. Data were searched against concatenated Uniprot human and BCG databases, and common contaminants (Cox et al., 2011). Fragment ion tolerance was set to 0.5 Da with a maximum of 2 missed tryptic cleavage sites. Carbamidomethyl cysteine was defined as a fixed modification, oxidized methionine, N-terminal acetylation and deamidation (NQ) were selected as variable modifications. Reversed decoy databases were used and the false discovery rate for both peptides and proteins were set at 0.01. Protein quantitation utilized razor and unique peptides and required a minimum of 2 ratio counts and normalized protein ratios reported. Peptide re-quantify was enabled in all analyses apart from where indicated. Significance B values were calculated and Gene Ontology Cellular Compartment (GOCC) terms added using Perseus version 1.2.0.16^1^.

To assess the number of PM proteins identified, we summed the proteins with GOCC terms PM, cell surface but not PM (CS) and extracellular but not PM/CS (XC) as previously described (Weekes et al., 2010, 2012). We previously identified a subset of proteins annotated by GO as integral to the membrane, but with no subcellular assignment and a short GOCC (ShG) term (a 4-part term containing the terms ‘integral to membrane’ ‘intrinsic to membrane’, ‘membrane part’, ‘cell part’ or a 5-part term containing ‘membrane’ in addition to these terms) (Weekes et al., 2010, 2012). Where a majority of proteins identified from a given sample are annotated PM/CS/XC, it is likely that a substantial proportion of the proteins with a short GOCC term are also integral PM proteins – including these proteins provides a useful upper estimate of the number of identified PM proteins. To generate a list of proteins quantified with high confidence, we extracted all proteins quantified in both Experiments A and B. All such proteins were similarly modulated in both independent biological repeats.

### Flow Cytometry

THP-1 monocytes were differentiated into macrophages as described above, and infected with BCG-GFP at an MOI of 5:1 for 4 h. Non-phagocytosed bacteria were washed away as described above. Cells were harvested at 48 h post-infection and labeled with antibodies prior to analysis by flow cytometry. The degree of up- or down-regulation of a protein was calculated by comparing the mean fluorescent intensity of infected (FL-1^HI^ macrophages that had phagocytosed green BCG) macrophages with uninfected macrophages.

### Antibodies

Antibodies used for surface staining for flow cytometry were: anti-ABCA1 (clone AB.H10), anti-CD14-APC (clone 61D3) and anti-SLAMF7-PE (clone 162) (from: eBioscience, Inc, CA, USA); and goat anti-mouse-APC (Invitrogen, NY, USA).

### Flow Cytometry Analysis

Cells were washed away from plates and stained in PBS with 5% FBS for 30 min at 4C using the monoclonal antibodies listed above. Cells which stained by unlabeled antibody were then washed by PBS for 2 times and stained in PBS with 5% FBS for 30 min at 4C using Alexa Fluor488, Alexa Fluor405 or APC labeled secondary antibody (from Invitrogen). Data were acquired on an Accuri^TM^*C6* (BD) or a MACSQuant^®^ VYB (Miltenyi Biotec) and analyzed using FlowJo software.

### Mycobacterial Infection Protocols

THP-1 cells were activated and differentiated into adherent macrophages by 16 μM phorbol-12-myristate-13-acetate (PMA) 48 h prior to infection. Bacterial cultures in logarithmic growth phase were centrifuged, washed, resuspended with RPMI-1640 culture medium with 10% FBS, and filtered through a 5 μm filter to get single cell bacteria. Macrophages were infected with BCG (MOI of 5:1) for 7 h or *M. abscessus* (MOI of 5:1) for 4 h or Mtb (MOI of 10:1) for 4 h at 37°C with 5% CO2. Then, cells were washed twice with PBS and cultured in the presence of Amikacin (200 μg/ml) for 1 h to kill extracellular mycobacteria. Cells were washed three times using warm PBS to remove Amikacin and then fresh RPMI-1640 with 10% FBS and antibiotics (penicillin and streptomycin) were added to cells. Simvastatin – 10uM – (Sigma) was added to activated THP-1 cells 16 h prior to infection and washed away prior to infection and fresh simvastatin and/or mevalonate (100 μM, Sigma) added in fresh medium following infection.

### Colony Forming Unit (CFU) Determination

Virulent Mtb (H37Rv) or *M. abscessus* was grown to logarithmic growth phase in Middlebrook 7H9 broth (BD) with BBL Middlebrook OADC Enrichment (BD) and 0.05% Tween 80 (Sigma). After 4 h infection, infected macrophages were lysed at 0 h, 20 h, or 44 h incubation. Mycobacteria enumerated by plating serial dilutions of cell lysates on Middlebrook 7H10 agar plates. Briefly, infected macrophage were lysed with 1% Triton X-100 for 5 min and 10-fold dilutions were performed using 0.05% Tween 80 in PBS before plating on Middlebrook 7H10 (BD) supplemented with 10% oleic acid-albumin-dextrose-catalase enrichment (Difco). Colonies were counted after 21 days (Mtb) or 3 days (*M. abscessus*) of growth at 37°C. Survival rate was calculated as the percentage survival of bacteria (as enumerated by CFU) at time = X compared with lysis and plating immediately following infection.

### Lentivirus Preparation and Infection

Expression vector construction: the RNA interference sequence was designed as described (Fukuchi et al., 2004). DNA coding for an RNAi for human ABCA1 was prepared with the following oligonucleotides: 5-GATCCCCTGAGTTTAGGTATGGCGGCTTCAAGAGA-3 and 5-TCGAGAAAAATGAGTTTAGGTATGGCGGCTCTCTTGAA-3. These oligonucleotides were annealed and ligated into the pEN_H1 vector. Using the Gateway^®^ LR recombination reaction, the RNAi sequence was shuttled into a large expression vector pDSL_hpUGIP.

To package lentivirus: HEK293T cells were seeded 24 h before transfection. PDSL_hpUGIP or PDSL_hpUGIP contained RNAi sequence, pMD2.G and psPAX2 were transfected into HEK293T cells using Lipofectamine^®^ LTX with Plus^TM^ (from Invitrogen) following manufacturer’s instruction. Reagents without plasmid were added into HEK293T as a negative control. Forty-eight hours after transfection, the supernatants were collected and filtrated with 0.45 μm filter.

To infect THP-1 cells: fresh RPIM-1640 with 10% FBS was mix with the filtrated supernatant at 2 to 1 ratio and add to THP-1 cells. Twenty-four hours after infection, cells were collected and resuspended with fresh RPIM-1640 with 10% FBS. The expression of ABCA1 was detected by flow-cytometry 96 h after infection.

### Statistical Analysis

Statistical significance between groups was determined by unpaired two-tailed Student’s *t*-test. ^∗^*p* < 0.05, ^∗∗^*p* < 0.01.

## Results

### Infection of Human Monocyte THP1 Cells with BCG Causes Dramatic Changes in Expression of Plasma Membrane Proteins

**FIGURE 1:**
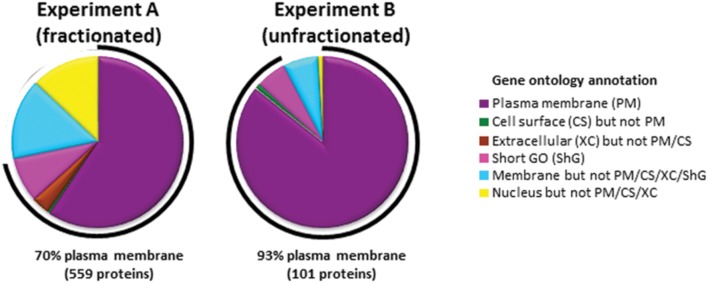
**Gene ontology annotations of proteins identified by two or more peptides in each experiment.** In Experiment A, peptides were fractionated by HpRp, and in Experiment B (label swap) unfractionated. Short GO terms include the annotations ‘membrane’, ‘integral to membrane’, ‘intrinsic to membrane’, ‘cell part’, but have no information as to subcellular localization.

**FIGURE 2:**
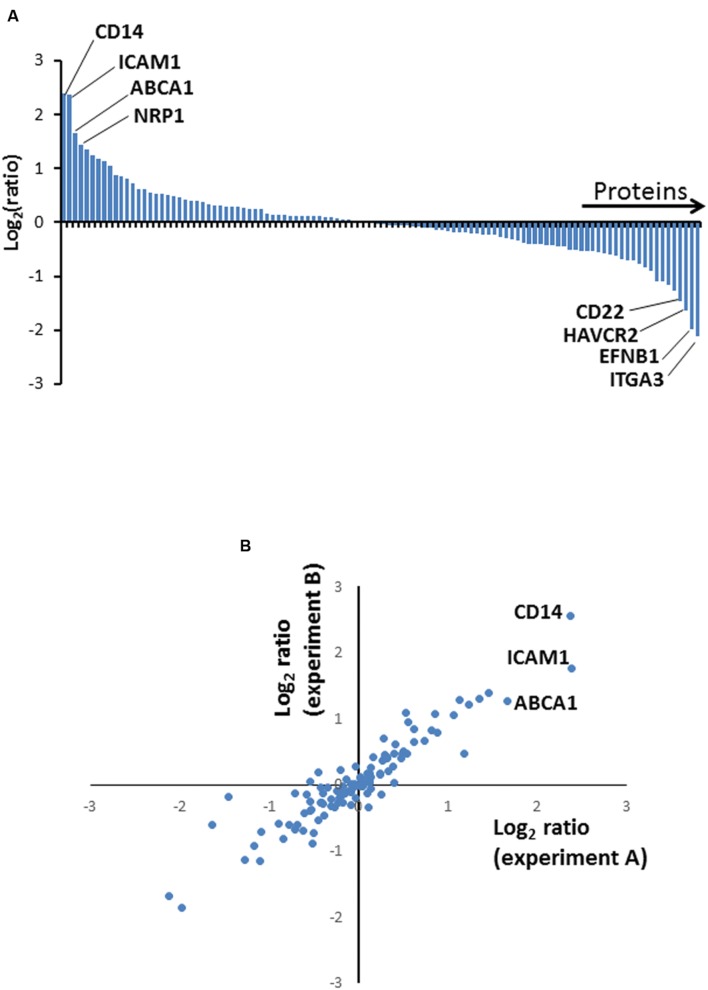
**(A)** Column chart illustrating high confidence proteins quantified in two independent biological repeat experiments. Ratios are shown as log_2_, and are illustrated from Experiment A. **(B)** Correspondence between protein ratios from Experiments A and B. Ratios are shown as log_2_.

To identify cell surface proteins modulated by BCG infection, we used ‘plasma membrane profiling’ to compare infected with control cells. We fractionated peptides using high pH reversed phase liquid chromatography to increase the number of proteins quantified. We quantified 873 proteins, of which 559 had a Gene Ontology annotation ‘plasma membrane’ (PM), ‘cell surface’ (CS), ‘extracellular’ (XC), or ‘short GO’ (ShG, see methods) (70% of all quantified proteins with a GO annotation; Experiment A). To ensure all light-labeled skin contaminants were fully excluded, a ‘label swap’ unfractionated sample was also analyzed which identified 110 proteins, of which 93% (101 proteins) were annotated PM/CS/XC/ShG (Experiment B, **Figure 1**). We considered the overlap between Experiments A and B ‘high confidence’ data, which is displayed in **Figures 2A,B**; **Supplementary Table S1**. Full data is shown in **Supplementary Table S2**.

**FIGURE 3:**
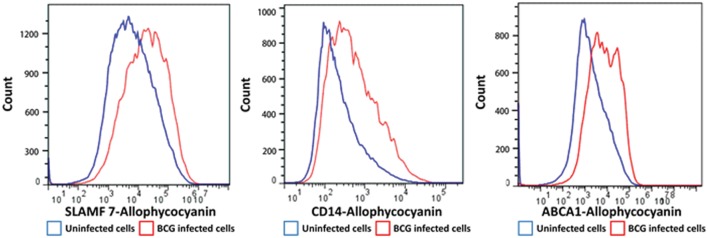
**Flow cytometry histograms of a selection of proteins with altered cell surface expression following BCG infection of THP-1 macrophages.** Macrophages were infected with BCG-GFP (MOI: 5:1) for 4 h and then analyzed 48 h following infection by flow cytometry. Infected macrophages (GFP^HI^) and uninfected macrophages (GFP^LO^) were compared by mean fluorescent intensity (MFI).

We selected a subset of PM proteins whose expression was significantly affected by BCG infection and for which commercially available antibodies were available for flow cytometry (**Figure 3**). We confirmed upregulation of the most significantly upregulated high confidence proteins CD14 and ATP-binding cassette sub-family A member 1 (ABCA1) (**Figures 2** and **3**), in addition to SLAM Family Member 7 (SLAMF7) (**Figure 3**; **Supplementary Table S2**).

### Canonical Pathway Analysis Suggests BCG Infection Modulates Immune Interactions and Lipid Metabolism

In order to identify pathways that were modulated upon BCG infection, we performed Ingenuity Pathways Analysis^2^ (Ingenuity^®^ Systems, Mountain View, CA, USA) on all proteins up- or down-regulated >2.5-fold. In order to increase the ability of this analysis to detect regulated pathways, we used the full set of up- or down-regulated proteins derived from Experiment A. Multiple functional terms suggest an effect of BCG infection on host immunity, including ‘lipid metabolism’, ‘Immune Cell Trafficking’, ‘Inflammatory Response’, ‘Antigen Presentation’ and ‘Cell-mediated Immune Response’. Pathways significantly modulated by BCG infection include CD40 signaling, and leukocyte extravasation signaling as well as LXR/RXR activation. The LXR/RXR pathway is linked to lipid metabolism in macrophages (A-González and Castrillo, 2011).

### Numerous Cholesterol-Associated Receptors Are Differentially Regulated upon Macrophage Infection

It has been suggested that although mycobacteria are able to metabolize a wide variety of carbon sources *in vitro*, the key carbon source *in vivo* is likely to be cholesterol (Pandey and Sassetti, 2008; Griffin et al., 2011, 2012; Ouellet et al., 2011). We noted with interest, therefore, that several proteins highlighted by Ingenuity analysis of ‘lipid metabolism’ pathways were implicated in cholesterol metabolism and inflammation and were differentially expressed on the PM following BCG-infection. Upregulated proteins included Zinc alpha 2-glycoprotein (Ceperuelo-Mallafre et al., 2012), Low Density Lipoprotein (LDL) Receptor, CD82 (Delaguillaumie et al., 2004), CXCR7 (Li et al., 2014), and ATP-binding cassette subfamily A Member 1 (ABCA1) (Ye et al., 2011). Downregulated proteins included Plasminogen activator inhibitor 1 RNA-binding protein (Kudo et al., 2004), Sortilin 1 and the Sortilin-related receptor (Strong et al., 2014), Endothelial cell-selective adhesion molecule (Inoue et al., 2010), and Ephrin B1 with its receptor (Lee and Daar, 2009).

### ABCA1 Upregulation Following Mycobacterial Infection Restricts Mycobacterial Growth

**FIGURE 4:**
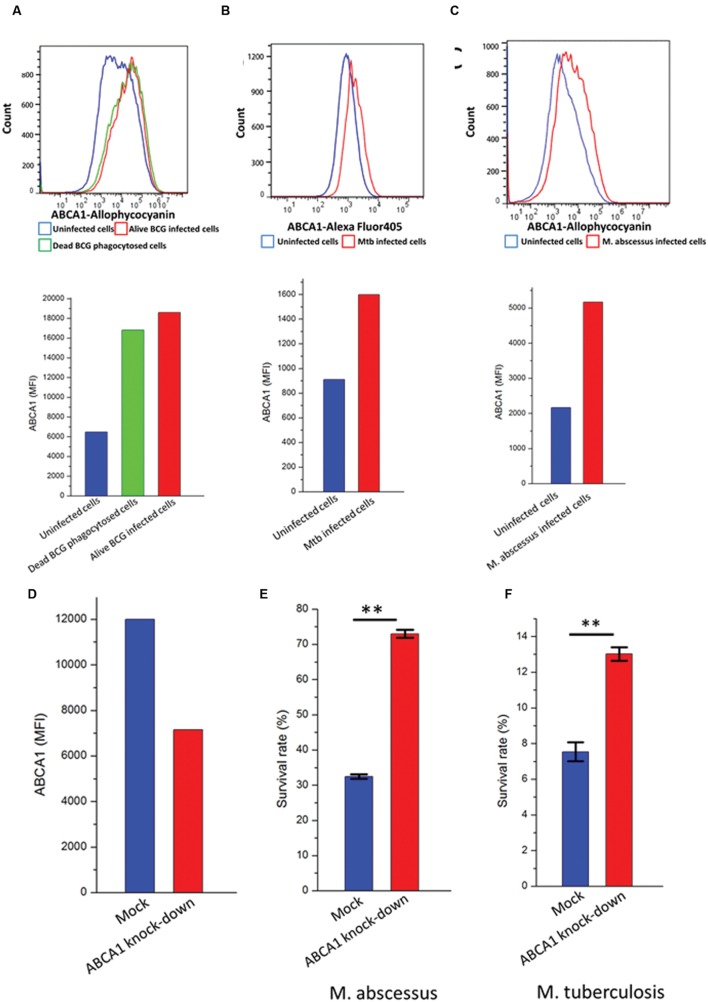
**ABCA1 is upregulated in response to mycobacterial infection and is a mycobacterial restriction factor.** Flow cytometry histograms (left) and graph (right) showing cell-surface ABCA1 staining of THP-1 macrophages infected by **(A)** fluorescently labeled live or heat-killed BCG (MOI: 5:1), **(B)** *M. tuberculosis*-H37Rv-GFP (MOI: 10:1), **(C)** *M. abscessus*-GFP (MOI: 5:1). **(D)** Relative cell-surface expression of ABCA1 on THP-1 macrophages following lentiviral-mediated knock-down as measured by flow cytometry. **(E)** Relative survival of *M. abscessus* following infection of THP-1 macrophages after 44 h following mock or ABCA1 knockdown. **(F)** Relative survival of *M. tuberculosis*-H37Rv following infection of THP-1 macrophages after 24 h following mock or ABCA1 knockdown. ^∗∗^*p* < 0.01 by Student’s *t*-test.

The cholesterol transporter ABCA1 pumps cholesterol from inside the cell to the outside (Jin et al., 2015) and may therefore alter the cellular content of cholesterol available to mycobacteria. We wished to investigate the functional role for upregulation of ABCA1 on mycobacterial infection. We noted a robust upregulation of ABCA1 following stimulation of activated THP-1 cells by both live and heat-killed BCG (**Figure 4A**). This was suggestive of a host response to mycobacterial infection. To verify that ABCA1 upregulation was not a generalized response to particle phagocytosis we fed activated THP-1 macrophages with fluorescently labeled beads. Phagocytosis of the beads was associated with a small down-regulation of surface ABCA1 expression (**Supplementary Figure S1**), suggesting that ABCA1 upregulation was a specific response to stimulation by mycobacterial components.

Our proteomic experiments were performed on BCG, a relatively non-pathogenic relative of pathogenic Mtb. We wished to extend the implications of our data to pathogenic mycobacteria such as *M. tuberculosis* and *M. abscessus*. Previous reports had noted upregulation of ABCA1 gene expression in murine macrophages infected by *M. tuberculosis*-H37Rv (Stavrum et al., 2012), but not examined ABCA1 protein expression. We verified that infection of activated THP-1 cells, a human monocytic cell-line by pathogenic mycobacteria such as *M. tuberculosis*-H37Rv (**Figure 4B**) and *M. abscessus* (**Figure 4C**) also led to increased plasma-membrane expression of ABCA1.

To determine whether ABCA1 upregulation benefits pathogen or host, we knocked-down ABCA1 expression by RNA interference. Lentiviral transduction of activated THP-1 cells with short-hairpin RNA (shRNA) targeting ABCA1 led to decreased cell-surface expression of ABCA1 (**Figure 4D**). This knockdown led to increased survival of intra-cellular *M. tuberculosis* (**Figure 4E**) and *M. abscessus* (**Figure 4F**) and this was not due to differences in infection rate in macrophages where ABCA1 was knocked down (**Supplementary Figure S2**).

**FIGURE 5:**
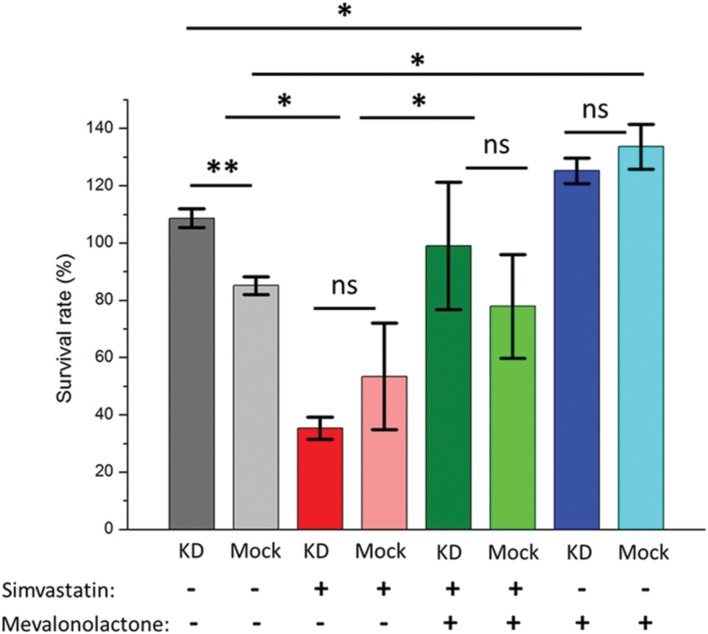
**Pharmacological depletion of cholesterol in THP-1 macrophages results in decreased intra-cellular mycobacterial survival.** Activated macrophages were pre-treated with simvastatin or mevalonate or both and infected with *M. abscessus* (MOI 5:1). Survival of intra-cellular bacteria was determined 44 h following infection compared with immediately following phagocytosis (hence survival rates >100%) – see Materials and Methods. KD = ABCA1 knock-down by lentiviral transduction, Mock = empty vector. ^∗^*p* < 0.05, ^∗∗^*p* < 0.01 by Student’s *t*-test.

To determine whether the phenotype associated with ABCA1 depletion was due to its effects on cellular cholesterol depletion, we used the pharmacological agents simvastatin and mevalonate (Parihar et al., 2013). Mevalonate is a water-soluble precursor of cholesterol and its synthesis by HMG-CoA reductase is inhibited by simvastatin. Simvastatin depletes cellular cholesterol stores, which would be rescued by the addition of mevalonate, and simvastatin treatment has recently been shown to potentiate TB treatment (Dutta et al., 2016). Treating THP-1 cells infected with *M. abscessus* with simvastatin following knock-down of ABCA1 or empty vector led to a substantial decrease in mycobacterial survival (**Figure 5**), with no difference in mock-treated or ABCA1 knock-down cells. This suggested that, with the cellular depletion of cholesterol by simvastatin, ABCA1 inhibition had no additive effect. Whereas addition of mevalonate rescued the simvastatin phenotype and mevalonate alone increased intra-cellular mycobacterial survival, regardless of ABCA1 knock-down (**Figure 5**). Taken together, these data are highly suggestive that ABCA1 upregulation following mycobacterial infection is a host response to restrict mycobacterial growth by intra-cellular cholesterol depletion.

## Discussion

Our study provides the first application of PMP to investigate quantitative changes to PM proteins following bacterial infection, in this case *M. bovis*-BCG. PMP has until now been applied to quantify cell surface proteins modulated by individual host or viral genes (Weekes et al., 2012, 2013) or viral infection (Weekes et al., 2014). Previous proteomic studies of mycobacterial host-pathogen interactions have studied whole-cell and phagosomal proteomes in response to purified mycobacterial components (Shui et al., 2009, 2011). Infection of host cells by mycobacteria have investigated cytosolic and nuclear proteomes (Wang et al., 2013), phagosomes (Rao et al., 2009; Lee et al., 2010; Li et al., 2011; Singh et al., 2011), mitochondria (Jamwal et al., 2013), and endoplasmic-reticulum proteomes (Saquib et al., 2015). This is the first study examining specific changes in PM proteins following mycobacterial infection.

Our analysis highlighted a number of coordinated changes in surface expression of host proteins, including cell adhesion (integrin alpha-1, -3, and -7 and ICAM1), innate immunity (CD14 and CD40) and cholesterol metabolism (ABCA1, zinc alpha 2-glycoprotein, LDL-R and CD82). We focused on upregulation of ABCA1 for further functional investigation due to the proposed critical role of cholesterol as a host-derived carbon source following tuberculosis infection.

The first indication that pathogenic *M. tuberculosis* and its close relative BCG can utilize cholesterol as a major nutrient both *in vitro* and in infection of mice and murine macrophages came from the groups of Eltis and Sassetti (Van der Geize et al., 2007; Pandey and Sassetti, 2008). Sassetti and Pandey showed that mutants of *M. tuberculosis* lacking a cholesterol importer, Mce4, were highly attenuated in the chronic persistence phase of murine infection or in activated macrophages (Pandey and Sassetti, 2008), although cholesterol utilization did not appear to be essential in a guinea-pig model of infection (Yang et al., 2011). Utilization of cholesterol as a carbon source results in a very specific program of gene expression and is conditionally essential, compared with growth of *M. tuberculosis* with ‘standard’ laboratory media (Griffin et al., 2011, 2012). Specifically inhibiting cholesterol degradation by mycobacteria using novel small molecule probes restricted growth in host cells (VanderVen et al., 2015), verifying the importance of cholesterol utilization by mycobacteria during infection.

Macrophages have a variety of mechanisms for regulation of cellular cholesterol and lipid content. The cholesterol transporters ABCA1 and ABCG1 export cholesterol from within macrophages to the extra-cellular milieu (Jin et al., 2015). ABCA1 is at least partially positively regulated by the liver × receptor LXR (Ito et al., 2015) and negatively regulated by the microRNA miR-33 (Rayner et al., 2010).

Regulation of ABCA1 has been previously shown to have divergent effects on pathogen survival. HIV specifically down-regulates surface expression of ABCA1 via Nef (Lin et al., 2015), and upregulation of ABCA1 by all-trans retinoic acid (ATRA) or LXRs restricts intra-cellular HIV replication (Jiang et al., 2012). Similarly, upregulation of ABCA1 inhibits hepatitis C virus (HCV) replication (Bocchetta et al., 2014). However, when investigating the intracellular bacterium *Listeria monocytogenes*, deletion of ABCA1 in the myeloid lineage was protective in a murine model of pathogenesis (Zhu et al., 2012). Unlike our data which supports upregulation of ABCA1 following mycobacterial infection (**Figures 1**–**4**), infection of lung epithelial cells with another intracellular pathogen, *Chlamydia pneumoniae* specifically downregulated ABCA1 (Korhonen et al., 2013) via upregulation of miR-33 (Zhao et al., 2014) and this was associated with decreased chlamydial survival within the host cells (Korhonen et al., 2013). In a murine model of cerebral malaria, ABCA1 deletion resulted in significantly less pathology due to both decreased inflammation and microparticle circulation (Combes et al., 2005).

There are no prior direct studies of ABCA1 and mycobacterial infection. However, a study examining LXR-deficient mice observed that these mice were highly susceptible to infection by *M. tuberculosis* (Korf et al., 2009). Similarly, macrophages induced with ATRA, a known ABCA1 agonist, restricted mycobacterial growth (Wheelwright et al., 2014). However, given that ABCA1 mediates pleiotropic inflammatory responses (Ito et al., 2015), it is not clear whether these effects are mediated via cholesterol restriction or other, inflammatory pathways.

In a study by Mahajan et al. (2012), the relatively non-pathogenic strain of *M. tuberculosis*, H37Ra, induced downregulation of ABCA1 gene expression as measured by RT-PCR. Although we did not measure total ABCA1 expression in response to mycobacterial infection, we noted a robust cell-surface expression of ABCA1 in response to both non-pathogenic (BCG – **Supplementary Tables S1** and **S2**; **Figure 4A**) and pathogenic (*M. tuberculosis* H37Rv and *M. abscessus*, **Figures 4B,C**) mycobacterial infection. Both their study and ours support a role for cholesterol-restriction by modulation of ABCA1 expression resulting in decreased intra-cellular mycobacterial survival. However, their data suggest mycobacteria specifically down-regulate ABCA1 gene expression by an unknown mechanism, whereas our data supports ABCA1 upregulation as a host-mediated response. Our data showing upregulation of ABCA1 by macrophages pulsed with dead BCG (**Figure 4A**) support ABCA1 upregulation as a host inflammatory response to pathogen-related molecular patterns.

Our data do not conclusively demonstrate the mechanism by which ABCA1 upregulation restricts mycobacterial growth within macrophages. Although it is intriguing to speculate it is by export of a nutrient essential for mycobacterial growth, cholesterol, the fact that knockdown or deletion of ABCA1 also benefits *Listeria* (Zhu et al., 2012) – a pathogen not known to catabolise cholesterol, suggests that this may be a more generalized inflammatory response mediated by ABCA1 (Ito et al., 2015). Alternatively, the mycobacterial and *Listeria* phenotypes may be mediated by different mechanisms: mycobacterial restriction via depletion of cholesterol as a carbon source, and *Listeria* restriction by depletion of cholesterol in the phagosome membrane, which would limit the effectiveness of the cholesterol-dependent cytolysin listeriolysin O (Parihar et al., 2013). Our data using the pharmacological agents simvastatin and mevalonate support a role for the effects of ABCA1 upregulation to be mediated by its direct effects on intra-cellular cholesterol stores. Regardless of the precise molecular mechanism, identification of a novel host-restriction factor against intra-cellular mycobacterial survival opens avenues for new treatment modalities targeting the host.

## Author Contributions

BJ conceived of the project. RB, MW, RA, DS, JL, and BJ analyzed data. RB, MW, YZ, RA, JL, YD, DS, and BJ performed research. The manuscript was written by RB, MW, and BJ with contributions of all authors. All authors have given approval to the final version of the manuscript.

## Conflict of Interest Statement

The authors declare that the research was conducted in the absence of any commercial or financial relationships that could be construed as a potential conflict of interest.

## Footnotes

1 http://maxquant.org

2 www.ingenuity.com

## Supplementary Material

**FIGURE S1 | Phagocytosis of beads does not lead to upregulation of cell-surface ABCA1.** Flow cytometry histogram **(left)** and graph **(right)** of cell-surface ABCA1 expression on THP-1 macrophages fed fluorescently labeled beads.

**FIGURE S2 | ABCA1 knockdown does not alter phagocytosis of mycobacteria by THP-1 cells.** Activated THP-1 macrophages (gated population, **A**) transduced with lentivirus to knockdown ABCA1 (**B**, right panel) or empty vector (**B**, mock, left) were infected by BCG-mCherry. Macrophages that were infected with lentivirus expressed GFP (*y*-axis) and those infected by BCG were red (*x*-axis). Knock-down of ABCA1 did not alter phagocytosis rates (top right quadrant) of BCG compared with the mock-infected control.

**TABLE S1 | High confidence hits from plasma membrane profiling following BCG infection of THP-1 macrophages**. Proteins identified from both Experiments A and B.

**TABLE S2 | Plasma membrane profiling hits following BCG infection of THP-1 macrophages.** All hits from Experiment A – see Materials and Methods.

## References

[B1] A-González N. Castrillo A. (2011). Liver X receptors as regulators of macrophage inflammatory and metabolic pathways. *Biochim. Biophys. Acta* 1812 982–994. 10.1016/j.bbadis.2010.12.015 21193033

[B2] Bocchetta S. Maillard P. Yamamoto M. Gondeau C. Douam F. Lebreton S. (2014). Up-regulation of the ATP-binding cassette transporter A1 inhibits hepatitis C virus infection. *PLoS ONE* 9:e92140 10.1371/journal.pone.0092140 PMC3960176 24646941

[B3] Ceperuelo-Mallafre V. Escote X. Vilades C. Peraire J. Domingo P. Solano E. (2012). Zinc alpha-2 glycoprotein is implicated in dyslipidaemia in HIV-1-infected patients treated with antiretroviral drugs. *HIV Med.* 13 297–303. 10.1111/j.1468-1293.2011.00976.x 22256965

[B4] Comas I. Coscolla M. Luo T. Borrell S. Holt K. E. Kato-Maeda M. (2013). Out-of-Africa migration and Neolithic coexpansion of *Mycobacterium tuberculosis* with modern humans. *Nat. Genet.* 45 1176–1182. 10.1038/ng.2744 23995134 PMC3800747

[B5] Combes V. Coltel N. Alibert M. van Eck M. Raymond C. Juhan-Vague I. (2005). ABCA1 gene deletion protects against cerebral malaria: potential pathogenic role of microparticles in neuropathology. *Am. J. Pathol.* 166 295–302. 10.1016/S0002-9440(10)62253-5 15632021 PMC1602289

[B6] Cox J. Matic I. Hilger M. Nagaraj N. Selbach M. Olsen J. V. (2009). A practical guide to the MaxQuant computational platform for SILAC-based quantitative proteomics. *Nat. Protoc.* 4 698–705. 10.1038/nprot.2009.36 19373234

[B7] Cox J. Neuhauser N. Michalski A. Scheltema R. A. Olsen J. V. Mann M. (2011). Andromeda: a peptide search engine integrated into the MaxQuant environment. *J. Proteome Res.* 10 1794–1805. 10.1021/pr101065j 21254760

[B8] de Souza G. A. Fortuin S. Aguilar D. Pando R. H. McEvoy C. R. van Helden P. D. (2010). Using a label-free proteomics method to identify differentially abundant proteins in closely related hypo- and hypervirulent clinical *Mycobacterium tuberculosis* Beijing isolates. *Mol. Cell. Proteomics* 9 2414–2423. 10.1074/mcp.M900422-MCP200 20190197 PMC2984234

[B9] de Souza G. A. Wiker H. G. (2011). A proteomic view of mycobacteria. *Proteomics* 11 3118–3127. 10.1002/pmic.201100043 21726049

[B10] Delaguillaumie A. Harriague J. Kohanna S. Bismuth G. Rubinstein E. Seigneuret M. (2004). Tetraspanin CD82 controls the association of cholesterol-dependent microdomains with the actin cytoskeleton in T lymphocytes: relevance to co-stimulation. *J. Cell Sci.* 117(Pt 22), 5269–5282. 10.1242/jcs.01380 15454569

[B11] Dutta N. K. Bruiners N. Pinn M. L. Zimmerman M. D. Prideaux B. Dartois V. (2016). Statin adjunctive therapy shortens the duration of TB treatment in mice. *J. Antimicrob. Chemother.* 71 1570–1577. 10.1093/jac/dkw014 26903278 PMC5007636

[B12] Fukuchi J. Hiipakka R. A. Kokontis J. M. Hsu S. Ko A. L. Fitzgerald M. L. (2004). Androgenic suppression of ATP-binding cassette transporter A1 expression in LNCaP human prostate cancer cells. *Cancer Res.* 64 7682–7685. 10.1158/0008-5472.CAN-04-2647 15520169

[B13] Griffin J. E. Gawronski J. D. Dejesus M. A. Ioerger T. R. Akerley B. J. Sassetti C. M. (2011). High-resolution phenotypic profiling defines genes essential for mycobacterial growth and cholesterol catabolism. *PLoS Pathog.* 7:e1002251 10.1371/journal.ppat.1002251 PMC3182942 21980284

[B14] Griffin J. E. Pandey A. K. Gilmore S. A. Mizrahi V. McKinney J. D. Bertozzi C. R. (2012). Cholesterol catabolism by *Mycobacterium tuberculosis* requires transcriptional and metabolic adaptations. *Chem*. *Biol.* 19 218–227. 10.1016/j.chembiol.2011.12.016 PMC3292763 22365605

[B15] Inoue M. Ishida T. Yasuda T. Toh R. Hara T. Cangara H. M. (2010). Endothelial cell-selective adhesion molecule modulates atherosclerosis through plaque angiogenesis and monocyte-endothelial interaction. *Microvasc*. *Res.* 80 179–187. 10.1016/j.mvr.2010.04.005 20406651

[B16] Ito A. Hong C. Rong X. Zhu X. Tarling E. J. Hedde P. N. (2015). LXRs link metabolism to inflammation through Abca1-dependent regulation of membrane composition and TLR signaling. *Elife* 4:e08009 10.7554/eLife.08009 PMC4517437 26173179

[B17] Jamwal S. Midha M. K. Verma H. N. Basu A. Rao K. V. Manivel V. (2013). Characterizing virulence-specific perturbations in the mitochondrial function of macrophages infected with *Mycobacterium tuberculosis*. *Sci. Rep.* 3:1328 10.1038/srep01328 PMC3580321 23435464

[B18] Jiang H. Badralmaa Y. Yang J. Lempicki R. Hazen A. Natarajan V. (2012). Retinoic acid and liver X receptor agonist synergistically inhibit HIV infection in CD4+ T cells by up-regulating ABCA1-mediated cholesterol efflux. *Lipids Health Dis.* 11:69 10.1186/1476-511X-11-69 PMC3391983 22676378

[B19] Jin X. Freeman S. R. Vaisman B. Liu Y. Chang J. Varsano N. (2015). ABCA1 contributes to macrophage deposition of extracellular cholesterol. *J*. *Lipid Res.* 56 1720–1726. 10.1194/jlr.M060053 PMC4548776 26203076

[B20] Jungblut P. R. Schaible U. E. Mollenkopf H. J. Zimny-Arndt U. Raupach B. Mattow J. (1999). Comparative proteome analysis of *Mycobacterium tuberculosis* and Mycobacterium bovis BCG strains: towards functional genomics of microbial pathogens. *Mol*. *Microbiol.* 33 1103–1117. 10.1046/j.1365-2958.1999.01549.x 10510226

[B21] Korf H. Vander Beken S. Romano M. Steffensen K. R. Stijlemans B. Gustafsson J. A. (2009). Liver X receptors contribute to the protective immune response against *Mycobacterium tuberculosis* in mice. *J*. *Clin. Invest.* 119 1626–1637. 10.1172/JCI35288 PMC2689129 19436111

[B22] Korhonen J. T. Olkkonen V. M. Lahesmaa R. Puolakkainen M. (2013). ABC-cassette transporter 1 (ABCA1) expression in epithelial cells in *Chlamydia pneumoniae* infection. *Microb*. *Pathog.* 6 57–61. 10.1016/j.micpath.2013.05.006 23707398

[B23] Kudo T. Nakayama E. Suzuki S. Akiyama M. Shibata S. (2004). Cholesterol diet enhances daily rhythm of Pai-1 mRNA in the mouse liver. *Am*. *J. Physiol. Endocrinol. Metab.* 287 E644–E651. 10.1152/ajpendo.00095.2004 15361354

[B24] Lee B. Y. Jethwaney D. Schilling B. Clemens D. L. Gibson B. W. Horwitz M. A. (2010). The *Mycobacterium bovis* bacille Calmette-Guerin phagosome proteome. *Mol*. *Cell. Proteomics* 9 32–53. 10.1074/mcp.M900396-MCP200 PMC2808266 19815536

[B25] Lee H. S. Daar I. O. (2009). EphrinB reverse signaling in cell-cell adhesion: is it just par for the course? *Cell Adh. Migr.* 3 250–255. 10.4161/cam.3.3.8211 19276658 PMC2712803

[B26] Li Q. Singh C. R. Ma S. Price N. D. Jagannath C. (2011). Label-free proteomics and systems biology analysis of mycobacterial phagosomes in dendritic cells and macrophages. *J*. *Proteome Res.* 10 2425–2439. 10.1021/pr101245u PMC3090528 21413810

[B27] Li X. Zhu M. Penfold M. E. Koenen R. R. Thiemann A. Heyll K. (2014). Activation of CXCR7 limits atherosclerosis and improves hyperlipidemia by increasing cholesterol uptake in adipose tissue. *Circulation* 129 1244–1253. 10.1161/CIRCULATIONAHA.113.006840 24374972

[B28] Lin S. Nadeau P. E. Mergia A. (2015). HIV inhibits endothelial reverse cholesterol transport through impacting subcellular Caveolin-1 trafficking. *Retrovirology* 12:62 10.1186/s12977-015-0188-y PMC4501058 26169283

[B29] Mahajan S. Dkhar H. K. Chandra V. Dave S. Nanduri R. Janmeja A. K. (2012). *Mycobacterium tuberculosis* modulates macrophage lipid-sensing nuclear receptors PPARgamma and TR4 for survival. *J*. *Immunol.* 188 5593–5603. 10.4049/jimmunol.1103038 22544925

[B30] Ouellet H. Johnston J. B. de Montellano P. R. (2011). Cholesterol catabolism as a therapeutic target in *Mycobacterium tuberculosis*. *Trends Microbiol.* 19 530–539. 10.1016/j.tim.2011.07.009 21924910 PMC3205253

[B31] Pandey A. K. Sassetti C. M. (2008). Mycobacterial persistence requires the utilization of host cholesterol. *Proc. Natl. Acad. Sci. U.S.A.* 105 4376–4380. 10.1073/pnas.0711159105 18334639 PMC2393810

[B32] Parihar S. P. Guler R. Lang D. M. Suzuki H. Marais A. D. Brombacher F. (2013). Simvastatin enhances protection against *Listeria monocytogenes* infection in mice by counteracting *Listeria*-induced phagosomal escape. *PLoS ONE* 8:e75490 10.1371/journal.pone.0075490 PMC3782446 24086542

[B33] Rao P. K. Singh C. R. Jagannath C. Li Q. (2009). A systems biology approach to study the phagosomal proteome modulated by mycobacterial infections. *Int. J. Clin. Exp. Med.* 2 233–247. 19918316 PMC2773677

[B34] Rappsilber J. Mann M. Ishihama Y. (2007). Protocol for micro-purification, enrichment, pre-fractionation and storage of peptides for proteomics using StageTips. *Nat. Protoc.* 2 1896–1906. 10.1038/nprot.2007.261 17703201

[B35] Rayner K. J. Suarez Y. Davalos A. Parathath S. Fitzgerald M. L. Tamehiro N. (2010). MiR-33 contributes to the regulation of cholesterol homeostasis. *Science* 328 1570–1573. 10.1126/science.1189862 20466885 PMC3114628

[B36] Saquib N. M. Jamwal S. Midha M. K. Verma H. N. Manivel V. (2015). Quantitative proteomics and lipidomics analysis of endoplasmic reticulum of macrophage infected with *Mycobacterium tuberculosis*. *Int. J. Proteomics* 2015:270438 10.1155/2015/270438 PMC4345262 25785198

[B37] Schubert O. T. Ludwig C. Kogadeeva M. Zimmermann M. Rosenberger G. Gengenbacher M. (2015). Absolute proteome composition and dynamics during dormancy and resuscitation of *Mycobacterium tuberculosis*. *Cell Host Microbe* 18 96–108. 10.1016/j.chom.2015.06.001 26094805

[B38] Shui W. Gilmore S. A. Sheu L. Liu J. Keasling J. D. Bertozzi C. R. (2009). Quantitative proteomic profiling of host-pathogen interactions: the macrophage response to *Mycobacterium tuberculosis* lipids. *J*. *Proteome Res.* 8 282–289. 10.1021/pr800422e PMC2655317 19053526

[B39] Shui W. Petzold C. J. Redding A. Liu J. Pitcher A. Sheu L. (2011). Organelle membrane proteomics reveals differential influence of mycobacterial lipoglycans on macrophage phagosome maturation and autophagosome accumulation. *J*. *Proteome Res.* 10 339–348. 10.1021/pr100688h PMC3018347 21105745

[B40] Singh C. R. Bakhru P. Khan A. Li Q. B. Jagannath C. (2011). Cutting edge: nicastrin and related components of gamma-secretase generate a peptide epitope facilitating immune recognition of intracellular mycobacteria, through MHC class II-dependent priming of T cells. *J*. *Immunol.* 187 5495–5499. 10.4049/jimmunol.1100521 PMC3221744 22039303

[B41] Stanley S. A. Barczak A. K. Silvis M. R. Luo S. S. Sogi K. Vokes M. (2014). Identification of host-targeted small molecules that restrict intracellular *Mycobacterium tuberculosis* growth. *PLoS Pathog.* 10:e1003946 10.1371/journal.ppat.1003946 PMC3930586 24586159

[B42] Stavrum R. Valvatne H. Stavrum A. K. Riley L. W. Ulvestad E. Jonassen I. (2012). *Mycobacterium tuberculosis* Mce1 protein complex initiates rapid induction of transcription of genes involved in substrate trafficking. *Genes Immun.* 13 496–502. 10.1038/gene.2012.24 22695749

[B43] Stover C. K. de la Cruz V. F. Fuerst T. R. Burlein J. E. Benson L. A. Bennett L. T. (1991). New use of BCG for recombinant vaccines. *Nature* 351 456–460. 10.1038/351456a0 1904554

[B44] Strong A. Patel K. Rader D. J. (2014). Sortilin and lipoprotein metabolism: making sense out of complexity. *Curr*. *Opin. Lipidol.* 25 350–357. 10.1097/MOL.0000000000000110 PMC4565516 25101658

[B45] Van der Geize R. Yam K. Heuser T. Wilbrink M. H. Hara H. Anderton M. C. (2007). A gene cluster encoding cholesterol catabolism in a soil actinomycete provides insight into *Mycobacterium tuberculosis* survival in macrophages. *Proc*. *Natl. Acad. Sci. U.S.A.* 104 1947–1952. 10.1073/pnas.0605728104 PMC1794314 17264217

[B46] VanderVen B. C. Fahey R. J. Lee W. Liu Y. Abramovitch R. B. Memmott C. (2015). Novel inhibitors of cholesterol degradation in *Mycobacterium tuberculosis* reveal how the bacterium’s metabolism is constrained by the intracellular environment. *PLoS Pathog.* 11:e1004679 10.1371/journal.ppat.1004679 PMC4335503 25675247

[B47] Wang H. Dong D. Tang S. Chen X. Gao Q. (2013). PPE38 of Mycobacterium marinum triggers the cross-talk of multiple pathways involved in the host response, as revealed by subcellular quantitative proteomics. *J*. *Proteome Res.* 12 2055–2066. 10.1021/pr301017e PMC3646403 23514422

[B48] Weekes M. P. Antrobus R. Lill J. R. Duncan L. M. Hor S. Lehner P. J. (2010). Comparative analysis of techniques to purify plasma membrane proteins. *J. Biomol. Tech.* 21 108–115. 20808639 PMC2922835

[B49] Weekes M. P. Antrobus R. Talbot S. Hor S. Simecek N. Smith D. L. (2012). Proteomic plasma membrane profiling reveals an essential role for gp96 in the cell surface expression of LDLR family members, including the LDL receptor and LRP6. *J*. *Proteome Res.* 11 1475–1484. 10.1021/pr201135e PMC3292266 22292497

[B50] Weekes M. P. Tan S. Y. Poole E. Talbot S. Antrobus R. Smith D. L. (2013). Latency-associated degradation of the MRP1 drug transporter during latent human cytomegalovirus infection. *Science* 340 199–202. 10.1126/science.1235047 23580527 PMC3683642

[B51] Weekes M. P. Tomasec P. Huttlin E. L. Fielding C. A. Nusinow D. Stanton R. J. (2014). Quantitative temporal viromics: an approach to investigate host-pathogen interaction. *Cell* 157 1460–1472. 10.1016/j.cell.2014.04.028 24906157 PMC4048463

[B52] Wheelwright M. Kim E. W. Inkeles M. S. De Leon A. Pellegrini M. Krutzik S. R. (2014). All-trans retinoic acid-triggered antimicrobial activity against *Mycobacterium tuberculosis* is dependent on NPC2. *J*. *Immunol.* 192 2280–2290. 10.4049/jimmunol.1301686 PMC3954114 24501203

[B53] Wisniewski J. R. Zougman A. Nagaraj N. Mann M. (2009). Universal sample preparation method for proteome analysis. *Nat*. *Methods* 6 359–362. 10.1038/nmeth.1322 19377485

[B54] Yang X. Gao J. Smith I. Dubnau E. Sampson N. S. (2011). Cholesterol is not an essential source of nutrition for *Mycobacterium tuberculosis* during infection. *J*. *Bacteriol.* 193 1473–1476. 10.1128/JB.01210-10 PMC3067635 21257778

[B55] Ye D. Lammers B. Zhao Y. Meurs I. Van Berkel T. J. Van Eck M. (2011). ATP-binding cassette transporters A1 and G1, HDL metabolism, cholesterol efflux, and inflammation: important targets for the treatment of atherosclerosis. *Curr. Drug Targets* 12 647–660. 10.2174/138945011795378522 21039336

[B56] Zhao G. J. Mo Z. C. Tang S. L. Ouyang X. P. He P. P. Lv Y. C. (2014). *Chlamydia pneumoniae* negatively regulates ABCA1 expression via TLR2-Nuclear factor-kappa B and miR-33 pathways in THP-1 macrophage-derived foam cells. *Atherosclerosis* 235 519–525. 10.1016/j.atherosclerosis.2014.05.943 24953492

[B57] Zhu X. Westcott M. M. Bi X. Liu M. Gowdy K. M. Seo J. (2012). Myeloid cell-specific ABCA1 deletion protects mice from bacterial infection. *Circ*. *Res.* 111 1398–1409. 10.1161/CIRCRESAHA.112.269043 PMC3494814 22955730

[B58] Zumla A. Maeurer M. Host-Directed Therapies Network (Hdt-Net) Consortium (2015). Host-directed therapies for tackling multi-drug resistant tuberculosis: learning from the pasteur-bechamp debates. *Clin. Infect. Dis.* 61 1432–1438. 10.1093/cid/civ631 26219693

