# Supplementary figures and images for: Plasma Membrane Profiling Reveals Upregulation of ABCA1 by Infected Macrophages Leading to Restriction of Mycobacterial Growth
Source: Front Microbiol. 2016 Jul 12;7:1086. doi: 10.3389/fmicb.2016.01086 (PMC4940386; doi:10.3389/fmicb.2016.01086)

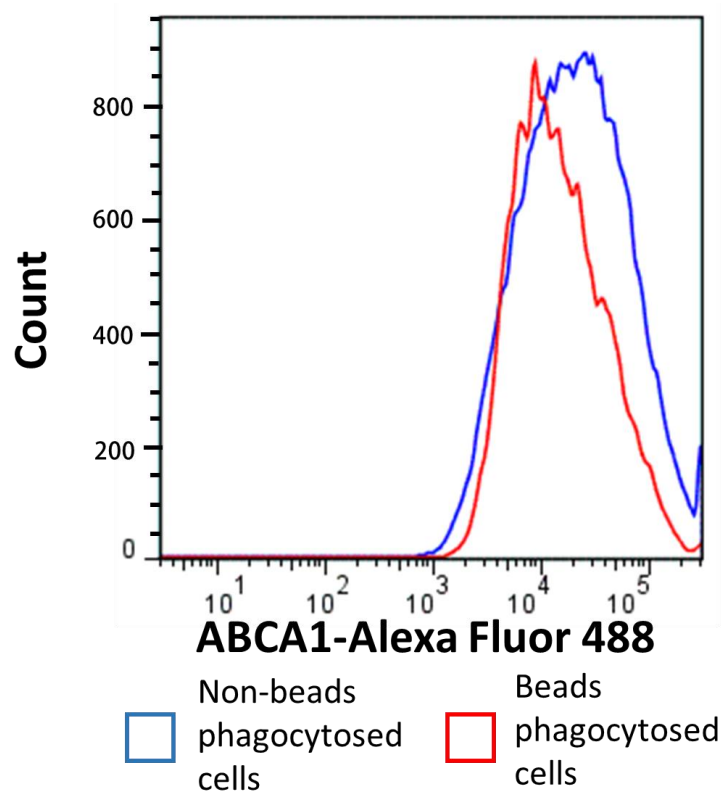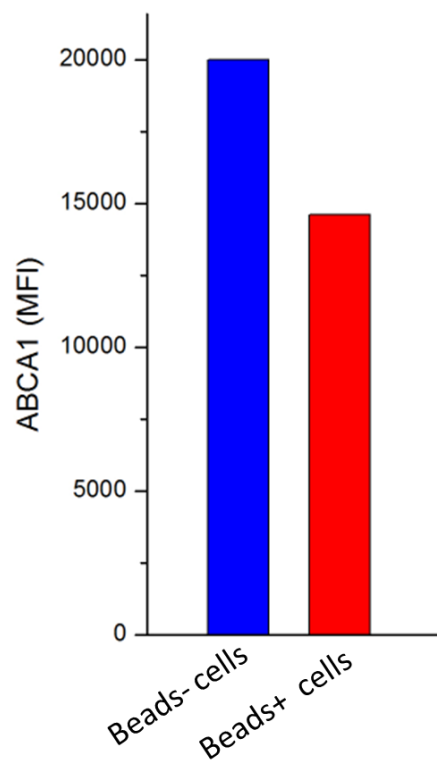

Figure S1

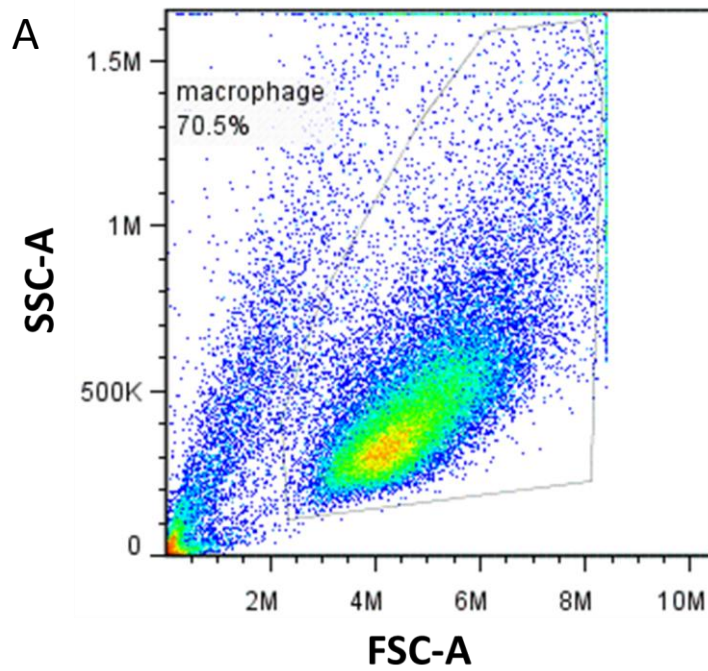

Total number of cells: 50000

Showed: 20% of total cells

**B**

**Empty vector (mock)**

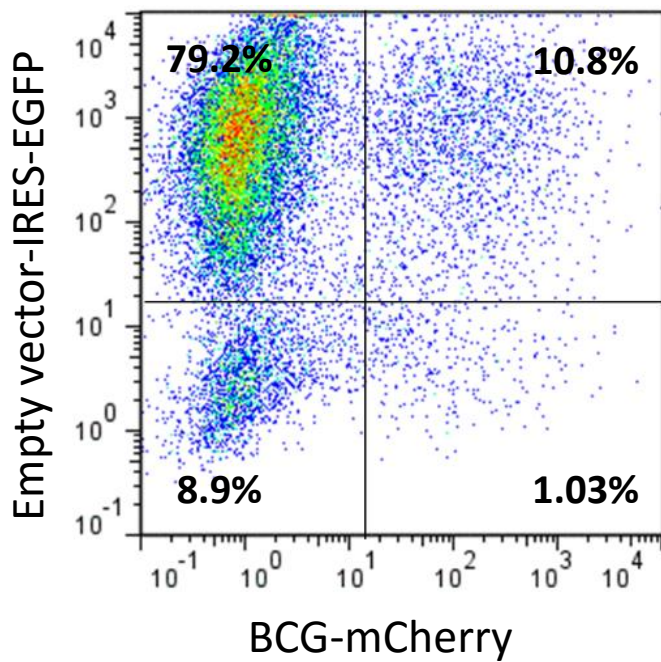

**ABCA1 shRNA**

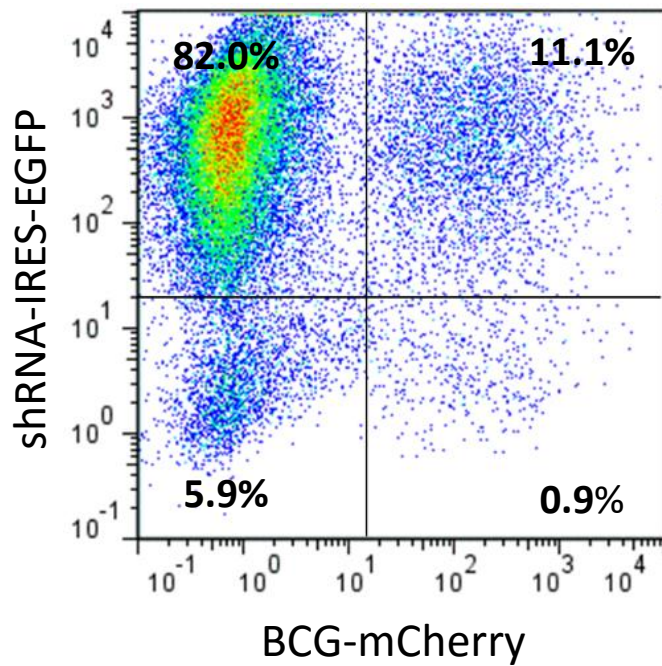

Figure S2

Supplement: Supplementary file 1 [file Image_1.PDF]
